# Prenatal telemedicine during COVID-19: patterns of use and barriers to access

**DOI:** 10.1093/jamiaopen/ooab116

**Published:** 2022-01-11

**Authors:** Allie Morgan, Daisy Goodman, Julia Vinagolu-Baur, Ilana Cass

**Affiliations:** 1 Geisel School of Medicine at Dartmouth, Hanover, New Hampshire, USA; 2 Department of Obstetrics and Gynecology, Dartmouth-Hitchcock Medical Center, Lebanon, New Hampshire, USA; 3 The Dartmouth Institute (TDI) for Health Policy and Clinical Practice, Hanover, New Hampshire, USA; 4 Division of Continuing Education, Harvard University, Cambridge, Massachusetts, USA

**Keywords:** obstetrics, telemedicine, pregnancy, communication, COVID-19, barriers to care

## Abstract

**Objective:**

To evaluate patient experience with a prenatal telemedicine visit and identify barriers to accessing telemedicine among rural pregnant people in northern New England during the beginning of the COVID-19 pandemic.

**Materials and Methods:**

We conducted a postvisit electronic survey of pregnant people who successfully participated in a prenatal telemedicine visit at a rural academic medical center in Northern New England. Nineteen questions were included in 5 domains: (1) engagement with prenatal care; (2) barriers to telemedicine and in person healthcare; (3) experience of prenatal care; (4) remote pregnancy surveillance tools; and (5) sources of COVID-19 information.

**Results:**

Responses were obtained from 164 pregnant people. Forty percent of participants had participated in an audio-only telemedicine visit, and 60% in a video telemedicine visit. The visit was easy or somewhat easy for 79% of respondents and somewhat difficult or difficult for 6.8%. The most common barrier to accessing telemedicine was poor internet or phone connectivity, followed by childcare responsibilities, lack of equipment, and lack of privacy. Participants also engaged in additional remote prenatal care including phone calls with registered nurses (7.6%), communication with the obstetrics team through a secure health messaging portal (21.1%), and home health monitoring (76.3%).

**Discussion and Conclusions:**

In this survey, evaluating the experience of pregnant people participating in a prenatal telemedicine visit during the COVID-19 pandemic, respondents had a positive experience with telemedicine overall, but also identified significant barriers to participation including issues with connectivity and lack of equipment for the visit. Most participants used telemedicine in combination with other tools for remote self-care.

## Lay Summary

To protect pregnant patients from infection during the COVID pandemic, maternity care providers turned to video and phone visits (“telemedicine”) to provide as much prenatal care as possible. To evaluate this change in our prenatal care program, we surveyed 164 pregnant people who had participated in a virtual prenatal visit about their care. Participants reported both positive and negative experiences, ranging from appreciation for having a safer option than in-person visits during the pandemic, to problems due to poor internet connection, lack of privacy, and lack of access to necessary equipment. Although 77.4% of respondents indicated they would recommend telemedicine to a friend, our program evaluation highlights the fact that the ability to participate in virtual care is not equally distributed. Unless steps are taken to address this problem, relying on telemedicine for a significant portion of prenatal care could result in widening disparities in prenatal care and outcomes. Policymakers and healthcare systems which provide telemedicine must address issues of access to technology and connectivity to avoid adding to maternal health disparities.

## INTRODUCTION

As the COVID-19 pandemic rages across the United States, the day-to-day functioning of health systems has been profoundly affected, not only by the need to care for large numbers of acutely ill people, but by the need to prevent infecting others. To protect the public as well as the healthcare workforce, many health systems rapidly transitioned to virtual formats for as much noncritical care as possible. In one of the largest and fastest paradigm shifts in the healthcare industry, a significant proportion of appointments were conducted via telemedicine, defined as the secure sharing of medical information between patients and healthcare providers in different locations utilizing a variety of information and communication technologies including video, audio-only, and asynchronous messaging platforms.^1^ This was an important transition for prenatal services as pregnant people represent an especially vulnerable population during viral outbreaks, ^2^^,^^3^ and emerging research suggests that COVID-19 infection during pregnancy is associated with comparatively high rates of severe maternal morbidity, intensive care admissions and mortality, as well as poor obstetric outcomes.^3^^,^^4^

Prior to the current pandemic, telemedicine had been introduced as a useful addition to prenatal care particularly for rural populations, with reassuring data on safety and acceptability.^5^^,^^6^ Although this delivery format reduces the inconvenience of attending in-person visits and maximizes compliance with social distancing protocols during a pandemic, reliance on telemedicine raises concerns about equity in access to care, especially for resource-limited populations. In 2019, 44% of low-income Americans lacked broadband internet service and 29% did not own a smartphone, creating significant challenges to accessing internet-based telemedicine services.^7^ In addition, low-income households are less likely to have a landline telephone and more likely to have a no-contract cell phone plan requiring the purchase of data packages with limited minutes of use to operate.^8^^,^^9^ Both of these factors decrease the likelihood of having a working phone or adequate minutes to participate in telemedicine^10^ and hinder engagement in services overall, including scheduling appointments and asynchronous digital communication such as messaging through a patient portal.

This lack of access to technology essential for telehealth is compounded for those living in rural areas.^11^ In New Hampshire, only 47% of people living in rural communities have access to adequate broadband service compared to 70% of people living in urban communities.^12^ Due to the rapid rollout of telemedicine in the context of the pandemic, barriers such as access to a working phone or the internet were not fully addressed, leaving low-resource and rural patients with urgent, significant barriers to connecting with their healthcare team. As preexisting social inequalities widened during COVID-19,^13^ the adverse economic impacts disproportionately affected the most vulnerable: low-income and rural families.^14^^,^^15^

Recognizing the urgent need to enable remote visits during the pandemic, and the potential challenges to video-based telemedicine, emergency authorization of telemedicine services included coverage of audio-only telemedicine visits on a time-limited basis. However, access to technology itself was not addressed by federal or state legislation, and significant barriers to equitable access for vulnerable pregnant people persist.^16^^,^^17^ We report the results of a survey of pregnant people in a rural region of northern New England who participated in telemedicine during the initial stages of the pandemic, highlighting patterns of telemedicine usage as well as challenges to accessing telemedicine services and overall experience with the technology. The survey was conducted to evaluate experience with the telemedicine program among pregnant patients, to understand gaps in accessing telemedicine, and to inform policies and programmatic change necessary to improve equitable access to telemedicine in our rural community.

## MATERIALS AND METHODS

Due to the COVID-19 pandemic, on March 24, 2020, in-person routine prenatal care was abruptly converted to a hybrid model at our medical center. Approximately half of routine visits were scheduled via telemedicine utilizing either a video platform through the electronic medical record or audio-only platforms through cell phones or landlines. In-person visits were planned only at weeks 12, 20, 28, 36, 38, and 40, with the remaining visits conducted via telemedicine. Pregnant people were encouraged to participate in video telemedicine visits, but were offered a choice between video or audio-only visits. We conducted a postvisit electronic survey of 164 pregnant people who had successfully participated in a telemedicine visit with an obstetric provider early in the course of the pandemic (April through October 2020). Respondents were all patients of the Ob/Gyn program at our academic medical center, which serves rural and remote rural communities in central New Hampshire and Vermont as defined by the U.S. Department of Agriculture Economic Research Service.

A universal survey link was generated through a secure REDCap^18^ database and sent via email to patients by scheduling staff within 2 weeks after a completed telemedicine encounter. The survey included 19 core questions encompassing 5 domains: (1) engagement with prenatal care; (2) barriers to telemedicine and in person healthcare; (3) experience of prenatal care and patient satisfaction; (4) home monitoring for pregnancy surveillance; and (5) sources of information regarding COVID-19 and pregnancy (Supplementary Appendix 1). To protect participant confidentiality, demographic data were collected anonymously from the electronic medical record and reported in summary only, including mean distance to the hospital, calculated from the county seat in the respondents’ county of residence. The survey was developed as part of an initiative to improve access and quality of telemedicine for obstetric patients, and the analysis of survey results was determined not to be Human Subjects Research by the Dartmouth-Hitchcock Institutional Review Board.

## RESULTS

One hundred and sixty-four pregnant patients responded to the postencounter survey, ranging in gestational age from early first trimester through 38 weeks, with a mean of 19 weeks (18% survey response rate). Forty percent of participants had participated in an audio-only telemedicine visit, and 60% in a video visit (Table  1).

**Table 1.** Characteristics of survey respondents (original)

**Demographic characteristics**	
**Age** in years, M (SD)	33 (5.3)
**Race and ethnicity,** *n* (%)	
White	146 (90.1%)
Not Hispanic or Latino	157 (96.9%)
**Payor**, *n* (%)	
Commercial insurance	131 (86.8%)
Medicaid	20 (13.2%)
**Distance from county of residency to medical center**, *n* (%)	
<50 miles	124 (76.6%)
50–100 miles	33 (20.1%)
100–500 miles	2 (1.2%)
>500 miles	5 (3.1%)
**Utilized public transportation for health care visits,** *n* (%)	3 (1.9%)
**Pregnancy characteristics**	
**Gestational age** in weeks, M (SD)	19 (10)
**Parity,** M (SD)	0.8 (0.5)
**Type of telemedicine visit**, *n* (%)	
Video	101 (61.6%)
Audio-only	63 (38.4%)

### Engagement with prenatal care

**Figure 1.**
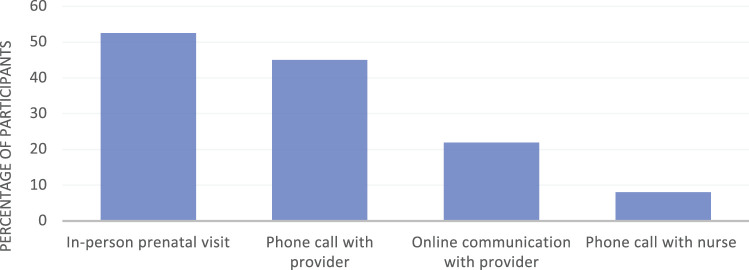
Type of interaction with OB team in the previous month.

The majority of respondents 52.6% (82/156) had an in-person prenatal visit within the last 4 weeks, 75.5% (114/151) had an in-person prenatal visit scheduled within the upcoming month, and 95.0% of respondents (150/158) reported some form of remote contact with the Obstetrics and Gynecology office within the 4 weeks prior to the survey, including 8.1% who had a phone call with a registered nurse, and 21.9% who had communicated with their provider team using a secure online electronic health portal. Fifteen percent (24/156) of respondents had not been seen in person at all during the current pregnancy. Only 16.1% (26/161) reported an unplanned in-person visit to address an urgent medical problem during this pregnancy (Figure  1).

### Pregnancy surveillance

Respondents reported having access to a variety of home self-monitoring tools or devices to augment remote visits although fewer reported using them (Table  2). The most popular self-monitoring tool was a thermometer (85%), followed by a scale (81%), blood pressure cuff (40.4%), and a Doppler device for auscultating the fetal heart rate (14.9%). In addition, 29.4% reported counting fetal movement (fetal kick counts) a no-cost, subjective assessment of fetal health.

**Table 2.** Respondent access to self-monitoring tools for pregnancy surveillance (original)

	**Respondents who reported owning the tool** (*n* = 141)	**Respondents who reported utilizing the tool** (*n* = 126)
**Thermometer**, *n* (%)	120 (85.1%)	53 (42.1%)
**Scale**, *n* (%)	114 (80.9%)	96 (76.2%)
**Blood pressure cuff**, *n* (%)	57 (40.4%)	38 (30.2%)
**Device for checking fetal heart rate**, *n* (%)	21 (14.9%)	14 (11.1%)
**Fetal kick counts**, *n* (%)		37 (29.4%)

### Experience of care

**Figure 2.**
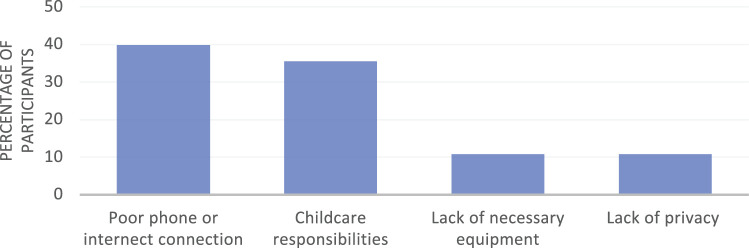
Patient-reported barriers to engaging in telemedicine (*n* = 98).

Seventy-nine percent of respondents (130/164) reported that their recent telemedicine visit was somewhat easy or very easy, whereas 6.8% (11/164) reported it was somewhat difficult or very difficult. Ninety-three survey respondents answered the survey question “are there things that make telemedicine visits hard?” (Figure  2). Of these, 39.8% cited poor internet or phone connectivity and 10.8% reported not having the right equipment. Additional challenges included lack of privacy and competing responsibilities, particularly childcare, as well as technological problems downloading required software. In free response fields, respondents had a range of impressions of the telemedicine experience, from frustration about not being able to connect with providers and disappointment at the lack of personal contact, through appreciation of the opportunity to have a remote visit rather than risking viral exposure or traveling long distances. In comparison, 69 respondents also identified challenges to attending in-person visits, including taking time off work (50.7%), childcare concerns (24.6%), access to transportation (7.2%), and money for gas (10.1%), as well as concern about exposure to SARS COV-2 through interaction with the healthcare system (7%). Despite some of the challenges identified with telemedicine, 77.4% of all respondents (127/164) would recommend telemedicine to a friend, while 17.1% (28/164) were unsure and only 5.5% (9/164) would not.

### Sources of information about risks of COVID-19 during pregnancy and lactation

During the first 9 months of the pandemic, there was a rapidly evolving body of evidence regarding the SARS CoV-2 virus and COVID-19 illness^4^^,^^19–22^ which made it challenging for pregnant people to stay up to date with regards to risks for themselves and their newborns. Concerns raised by respondents primarily focused on avoiding viral exposure generally, and specifically due to receiving prenatal care or during their delivery hospitalizations. Respondents also expressed anxiety about whether they would be able to have the support of a partner or other family members during childbirth. Nearly a quarter of these rural respondents (24%) reported that they lived or worked in circumstances where they could not adhere to recommended social distancing with people other than immediate family members.

Respondents reported obtaining information about the risks of COVID-19 during pregnancy and lactation from a variety of sources. The majority identified the internet sites as their primary source of information regarding COVID-19 and its potential impact on pregnancy and breastfeeding, 65.8% (96/146). These sources ranged from the World Health Organization and Centers for Disease Control websites, through popular pregnancy-specific sites like The Bump and What to Expect. Only 58.2% of participants (85/146) described medical providers as an important source of information and 30.1% (44/146) primarily obtained information about COVID-19 risks from friends and family. News media were the least frequently utilized, with 21.9% (32/146) and 13.7% (20/146) of participants reporting getting information from television and radio news sources, respectively.

## DISCUSSION

Our program evaluation confirmed the critical importance of facilitating equitable access to digital technology to protect the health and safety of pregnant people during the worldwide COVID-19 pandemic. Through this survey of pregnant people in rural northern New England during the initial stages of the COVID-19 pandemic, respondents described an overall positive experience, but also identified significant barriers to participation. Although most reported that the telemedicine system was somewhat or very easy to navigate, the most common barriers to accessing telemedicine were poor phone or internet connectivity, and more than 10% of respondents lacked access to necessary equipment. These barriers are likely to disproportionately impact both rural and low-income pregnant people.^8^^,^^9^^,^^11^ These findings are particularly concerning given the fact that the survey was deployed only to patients who had successfully participated in either an audio-only or video telemedicine visit, and highlight the reality that reliance on telemedicine can worsen disparities in access to healthcare.

Over a quarter of respondents noted that lack of childcare served as a barrier to engaging in a telemedicine visit, a finding consistent with recent studies describing the burden of childcare caused by pandemic-related school closures, daycare closures, quarantines, and shifts to home-based work on parents, and in particular, on women, causing many to leave the workforce.^15^^,^^23^^,^^24^ Together, inadequate internet access, poor phone connection, lack of equipment, and family responsibilities created barriers to accessing telemedicine for a sizeable proportion of our survey respondents. In addition to inequities in access to equipment and connectivity, respondents reported difficulty downloading software and navigating telemedicine visits, suggesting that patient-centered technical support is also necessary to address disparities in access. Of interest, more respondents relied on internet sources for health information than on information provided by healthcare providers, highlighting the importance of a multimedia approach for disseminating accurate information about COVID-19 risks and mitigation strategies.

Our program evaluation demonstrates that current efforts to expand telemedicine can potentially worsen disparities in maternity care access. Ensuring that this does not happen will require a coordinated public health-focused approach at multiple system levels, ranging from policy changes to interventions addressing technology access at the community and clinical services levels. At the policy level, the long-term goals of consistent rural broadband access and cell phone coverage will take years and significant federal funding to achieve. However, in the short term, policies which can be enacted immediately could have a large impact on improving equity in access to telemedicine services. Specifically, existing partial federal subsidies for broadband and/or mobile technology could be expanded to ensure adequate coverage of mobile technology to facilitate engagement in health care. Requiring state Medicaid and other insurance providers ensure that every beneficiary has a basic smart phone and adequate data package sufficient for telemedicine participation would go far to fill the connectivity gap. Mobile technologies also have distinct benefits over broadband for rural residents, as they can be utilized wherever a cell signal is available, rather than being tied to a specific location. In addition, mobility can also address issues of privacy and safety and access to care from multiple locations, all of which are important for pregnant people. Finally, extending coverage for audio-only visits past the end of the Public Health Emergency would further ensure access for patients without reliable internet service. From the perspective of financial sustainability, these policies can be justified in parallel to current programs which require Medicaid plans to cover travel to and from medical appointments,^25^ as a means to address financial and geographic disparities in accessing medical care.

This program evaluation has important limitations. Our survey was conducted with pregnant people who successfully engaged in at least 1 telemedicine visit, thereby excluding patients who lacked access to telemedicine entirely and resulting in an underrepresentation of telemedicine barriers. To this point, only 13% of respondents were insured through Medicaid programs, in contrast to an overall prenatal program rate of 30% who are Medicaid insured, indicating disproportionate representation of low-income patients within survey results. This could be the result of existing technology barriers precluding telehealth participation. In the future, it will be important to specifically survey patients who were unable or unwilling to engage in telemedicine, as they represent those patients who are most vulnerable to being left behind by telemedicine expansion. In addition, small sample size and relative homogeneity (largely white, commercially insured), and restriction to patients of 1 rural, northern New England program limit the applicability of our findings to other regions and populations. Research on barriers to telemedicine for perinatal care delivery is critically needed and should include samples representing geographic, racial, and socioeconomic diversity. Finally, this survey was deployed only once per patient relatively early in the course of the pandemic, and therefore was unable to capture long-term experience of telemedicine or improvement in access over time, which are both crucial for understanding the feasibility of telemedicine beyond the COVID-19 pandemic.

Nevertheless, this survey of telemedicine experience among pregnant people in northern New England during the early months of COVID-19 pandemic provides insight into patterns of technology usage for healthcare overall, experience of telemedicine as a modality, and barriers to accessing telemedicine services among a rural and remote rural population. The majority of respondents used technology to engage with their healthcare teams in a variety of ways, including patient portals and phone calls as well as formal telemedicine visits, and many also participated in self-monitoring to augment remote care. Clearly, telemedicine has provided a number of benefits for perinatal patients and providers during the COVID-19 pandemic and will likely remain an important component of obstetric care after the pandemic retreats. However, access to telemedicine is not equitably distributed, especially in remote rural populations, and our program evaluation underscores disturbing disparities in technology access even among telemedicine participants. Optimizing telemedicine for prenatal care must therefore include focused work to make this modality universally accessible. It is critical that inequity in access to telemedicine be addressed at the policy, practice, and individual patient levels, to avoid widening already profound socioeconomic disparities existing among rural pregnant populations. Health systems should recognize that telemedicine can only fulfill its promise if all patients have access to the tools necessary for participation.

## AUTHOR CONTRIBUTIONS

This manuscript has been read and approved by all the authors involved, the requirements for authorship have been met for each author and each author believes that the manuscript represents honest work.

## SUPPLEMENTARY MATERIAL

Supplementary material is available at *JAMIA Open* online.

## CONFLICT OF INTEREST STATEMENT

None declared.

## DATA AVAILABILITY

The data underlying this article cannot be shared publicly for the privacy of individuals that participated in the study. The data will be shared on reasonable request to the corresponding author.

## Supplementary Material

ooab116_Supplementary_Data
