# Supplementary material for: Prenatal telemedicine during COVID-19: patterns of use and barriers to access
Source: JAMIA Open. 2022 Jan 11;5(1):ooab116. doi: 10.1093/jamiaopen/ooab116 (PMC8822407; doi:10.1093/jamiaopen/ooab116)
Supplement: ooab116_Supplementary_Data [file ooab116_Supplementary_Data.pdf]

Appendix 1: Questions included in the telemedicine survey

1. What kind of telemedicine visit did you have recently?
    a. Phone, video, other
2. How easy or difficult was the telemedicine visit process?
    a. Very difficult, somewhat difficult, neither difficult nor easy, somewhat easy, very easy
3. Are there things that make telemedicine visits hard? (check all that apply)
    a. Don’t have the right equipment, poor phone or internet connection, lack of privacy, taking care of kids at the same time, other (please describe)
4. Are there things that make it hard to come in to the medical center for a visit? (Check all that apply)
    a. Transportation, childcare issues, money for gas, time off work, other (please describe)
5. Would you recommend telemedicine visits to a friend?
    a. Yes, no, not sure
6. Please tell us how far along you are in your pregnancy in weeks
7. When was your last in-person prenatal visit?
    a. 0 weeks ago, 1 week ago, 2 weeks ago, 3 weeks ago, 4 weeks ago, 5 weeks ago, 6 weeks ago, 7 weeks ago, 8 weeks ago, I haven’t had an in-person visit, I can’t remember
8. When is your next in-person prenatal visit?
    a. This week, 1 week from now, 2 weeks from now, 3 weeks from now, 4 weeks from now, >4 weeks from now, I don’t know
9. How long ago in weeks was the last phone call, text, myD-H or video contact with someone from the ObGyn office?
    a. This week, 1 week ago, 2 weeks ago, 3 weeks ago, 4 weeks ago, I don’t know, I can’t remember
10. What type of contact did you have?
    a. Phone call with registered nurse, phone call with doctor, nurse midwife, or nurse practitioner, text message with registered nurse, text message with community health worker, My-DH message, video visit, N/A
11. What went well about your most recent contact with our team?
12. What could be improved?
13. Have you needed to come in for an in-person visit to the office or Birthing Pavillion that you or your provider weren’t expecting?
    a. Yes, no
14. Do you have any of the following equipment at home? (check all that apply)
    a. Blood pressure cuff, scale, thermometer, device for checking the baby’s heartbeat
15. Are you doing any of the following activities at home? (Check all that apply)
    a. Checking blood pressure, kick counts, weighing yourself, taking your temperature, checking the baby’s heartbeat

16. Do you live or work in a place where you can be physically distant from people not in your immediate family?
    a. Yes, no
17. Do you need to carpool or use public transportation for work, shopping or health care visits?
    a. Yes, no
18. People get information about health from many sources. How have you learned about COVID-19 and how it might impact pregnancy and breastfeeding?
    a. The internet, television news, radio news, friends and family, medical providers, other (please share)
19. Is there anything you are particularly concerned about during the current public health emergency?
